# Epidemiology of *Plasmodium vivax* Malaria in Peru

**DOI:** 10.4269/ajtmh.16-0268

**Published:** 2016-12-28

**Authors:** Angel Rosas-Aguirre, Dionicia Gamboa, Paulo Manrique, Jan E. Conn, Marta Moreno, Andres G. Lescano, Juan F. Sanchez, Hugo Rodriguez, Hermann Silva, Alejandro Llanos-Cuentas, Joseph M. Vinetz

**Affiliations:** 1 Instituto de Medicina Tropical “Alexander von Humboldt,” Universidad Peruana Cayetano Heredia, Lima, Peru.; 2 Research Institute of Health and Society, Université Catholique de Louvain, Brussels, Belgium.; 3 Departamento de Ciencias Celulares y Moleculares, Facultad de Ciencias y Filosofia, Universidad Peruana Cayetano Heredia, Lima, Peru.; 4 Department of Biomedical Sciences, School of Public Health, University at Albany (State University of New York), Albany, New York.; 5 Wadsworth Center, New York State Department of Health, Albany, New York.; 6 Division of Infectious Diseases, Department of Medicine, University of California San Diego, San Diego, California.; 7 Facultad de Salud Pública, Universidad Peruana Cayetano Heredia, Lima, Peru.; 8 Dirección Regional de Salud Loreto, Ministerio de Salud, Iquitos, Peru.

## Abstract

Malaria in Peru, dominated by *Plasmodium vivax*, remains a public health problem. The 1990s saw newly epidemic malaria emerge, primarily in the Loreto Department in the Amazon region, including areas near to Iquitos, the capital city, but sporadic malaria transmission also occurred in the 1990s–2000s in both north-coastal Peru and the gold mining regions of southeastern Peru. Although a Global Fund-supported intervention (PAMAFRO, 2005–2010) was temporally associated with a decrease of malaria transmission, from 2012 to the present, both *P. vivax* and *Plasmodium falciparum* malaria cases have rapidly increased. The Peruvian Ministry of Health continues to provide artemesinin-based combination therapy for microscopy-confirmed cases of *P. falciparum* and chloroquine–primaquine for *P. vivax*. Malaria transmission continues in remote areas nonetheless, where the mobility of humans and parasites facilitates continued reintroduction outside of ongoing surveillance activities, which is critical to address for future malaria control and elimination efforts. Ongoing *P. vivax* research gaps in Peru include the following: identification of asymptomatic parasitemics, quantification of the contribution of patent and subpatent parasitemics to mosquito transmission, diagnosis of nonparasitemic hypnozoite carriers, and implementation of surveillance for potential emergence of chloroquine- and 8-aminoquinoline-resistant *P. vivax*. Clinical trials of tafenoquine in Peru have been promising, and glucose-6-phosphate dehydrogenase deficiency in the region has not been observed to be a limitation to its use. Larger-scale challenges for *P. vivax* (and malaria in general) in Peru include logistical difficulties in accessing remote riverine populations, consequences of government policy and poverty trends, and obtaining international funding for malaria control and elimination.

## Background

Peru, located along South America's central Pacific coast, is home to about 30 million inhabitants living in 1,838 municipalities in 24 administrative departments and one constitutional province (Callao, adjacent to Lima)^1^; the population is highly concentrated in the arid megacity, Lima, with almost 10 million inhabitants. Arthropod-borne diseases (malaria, arboviruses) generally are not important public health threats in Lima and the southern desert regions, but affect the humid, tropical regions where the environment sustains arthropod vectors. Peru has the third greatest biodiversity in the world, largely concentrated in the humid tropical ecosystems within 84 of the 117 recognized ecosystems found in Peru.^2^

**Figure 1.**
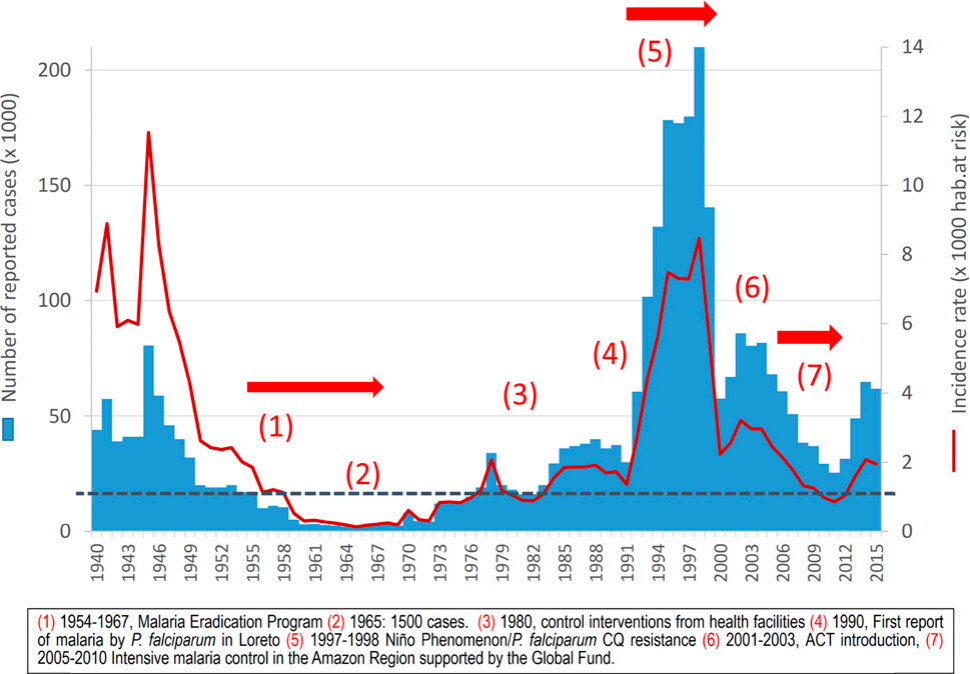
Historical trends of reported malaria incidence in Peru: 1940–2015. Source: Peruvian Ministry of Health.

Nationally, in 2015, 62,220 cases of malaria were reported in Peru,^3^ which accounts for about 15% of total reported malaria cases in the Americas and shows a continuing increase in cases since 2012 (Figures 1

and 2

**Figure 2.**
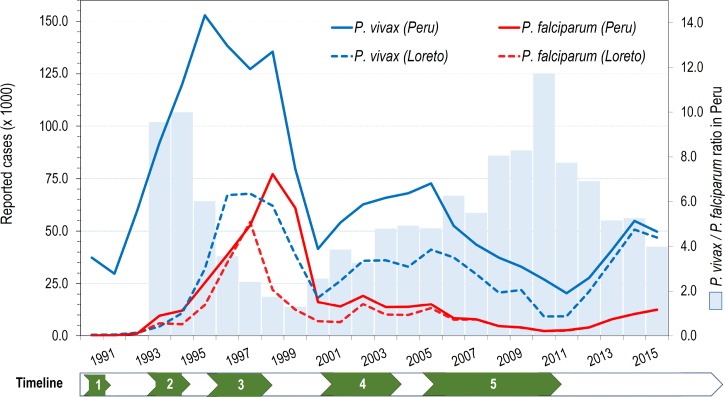
Annual reported cases by *Plasmodium* species in Peru and Loreto: 1990–2015. Information on annual malaria cases was obtained from the Regional Health Directorate of Loreto. Numbers in the timeline point out important events that influenced malaria incidence: 1) first report of *Plasmodium falciparum* in Loreto (1990); 2) first reports of chloroquine resistance (CQR) in *P. falciparum* (after 1994); 3) very strong El Niño Southern Oscillation (ENSO) phenomenon (1996–1998); 4) implementation of new antimalarial treatment policy for *P. falciparum* and *Plasmodium vivax* malaria (2001–2004); and 5) Global Fund-PAMAFRO project (2005–2010).

). Therefore, malaria remains an important public health problem in the country.^4^ In Peru, the vast majority of malaria is concentrated in Loreto Department (capital, Iquitos), in the northwest Amazon region, which accounts for 95% of reported malaria cases.^3^ Infection by *Plasmodium vivax* is more common than that by *Plasmodium falciparum* (Pv/Pf ratio of 4/1 in 2015). Malaria transmission is perennial but characterized by seasonal and epidemic increases of clinical cases detected by routine passive case detection (PCD) in some communities.^5^,^6^ Most affected people live in impoverished peri-urban and rural villages sparsely distributed along an extensive riverine network associated with tributaries feeding into the Amazon river.^6^,^7^ Intermittent epidemic malaria as well as sporadic cases take place in the Pacific northern coast,^8^ and transmission is low intensity in the high and central jungle,^9^ and the gold mining department of Madre de Dios,^5^ accounting together for about 5% of the total malaria cases reported in Peru.^3^

*Plasmodium vivax* malaria transmission is maintained in human populations by relapses from dormant liver parasite stages (i.e., hypnozoites),^10^ for which no diagnostic test exists^11^; treatment with primaquine (PQ) in Peru is given, but effectiveness is unclear because administration is not typically supervised.^12^ The presence of PQ resistance or compliance has not been systemically assessed under either routine or clinical trial conditions. Recent clinical trials of tafenoquine (TQ) (which included substantial enrollment of subjects in the Peruvian Amazon) for the treatment of acute *P. vivax* malaria have shown that this 8-aminoquinoline is effective not only for treatment and relapse in general,^13^ but also that there is a dose-dependent effect on symptomatic homologous strain relapse (but not heterologous strain reinfection).^14^ Subjects in these clinical trials were tested for glucose-6-phosphate dehydrogenase deficiency, but clinically significant low levels that would limit the use of 8-aminoquinolines were not found among more than 300 subjects. The increasing focal distribution of malaria transmission,^5^,^6^ the logistical difficulties in accessing remote riverine populations,^5^ the specific limitations for the control of *P. vivax* infections,^15^ together with the fact that a large proportion of those infections are asymptomatic and not detected by standard diagnostic methods such as light microscopy (LM),^5^,^6^,^16^,^17^ pose major challenges to the Peruvian national malaria control program (NMCP).

Despite the increasing research conducted in Peru over the past decade on *P. vivax* malaria epidemiology,^17^–^21^ diagnostics,^22^,^23^ treatment,^12^,^13^,^24^ vector biology,^25^–^28^ and molecular genetics,^29^–^31^ several knowledge gaps still need to be addressed to support the Peruvian NMCP, so that malaria transmission hotspots can be readily identified and that the NMCP can more effectively implement strategies that target such areas with effective interventions.^32^ In this review, the trends in *P. vivax* incidence are described in relation to major control efforts implemented in Peru over the past 70 years. The ecological context of *P. vivax*-endemic regions in Peru is described, and key research findings related to *P. vivax* transmission and its control in the country are outlined. Finally, the opportunities and challenges for not only controlling but also sustaining such measures toward *P. vivax* elimination are discussed.

## Persistence of *P. vivax* Transmission Despite Conventional Test and Treat Control Efforts Implemented by Public Health Agencies

Even though reported malaria cases were not systematically differentiated by *Plasmodium* species from the 1940s to the 1990s,^4^ it is likely that *P. vivax* dominated. Long-standing practices for gathering malaria case data are based on test-and-treat models, which involve Ministry of Health information systems that passively gather microscopy-confirmed malaria cases using paper registries. The data from these registries are reported and aggregated in a hierarchical fashion from health post to regional to national levels. Paper documentation is the practice at the local levels where the primary data are gathered. After peaking with over 80,000 cases in 1944,^4^ malaria dropped substantially in the following years; from 1960 to 1970, annual incidence rates remained below one case per 1,000 inhabitants with the lowest levels registered in 1965, when only about 1,500 total cases were reported (Figure 1).^33^ These results have been due to the use of dichloro-diphenyl-trichloroethane (DDT) in vector control campaigns after 1946, and to the shift of malaria control to a formal eradication strategy after 1957.^4^ The major vector in the Amazon (i.e., *Anopheles darlingi*) was nearly eliminated, and almost the entire coast, the inter-Andean valleys, and the Southern Amazon were determined to be free of malaria by 1970.^34^,^35^ However, the development of DDT resistance, as well as reduced political and public health interest resulting in decreased funding for control measures and the redirection of malaria control responsibilities toward test and treat strategies according to medical (i.e., treating individuals) not public health (population-based intervention) models, combined to halt progress in malaria control.^34^ It must be kept in mind that changes in governmental policies can lead to instability in the implementation of malaria control policies as well.

In the 1980s, the Peruvian NMCP was not structured to enable elimination activities.^34^ Following international guidelines, DDT use was first halted in the Peruvian Amazon in 1988, and then stopped across all of Peru. Predictably malaria reemerged in the 1990s, following the reintroduction and later spread of *An. darlingi* in the Amazon,^33^ as well as the simultaneous introduction of chloroquine (CQ)-resistant *P. falciparum* strains (CQ) in the northern coast^36^ and CQ and sulfadoxine–pyrimethamine (SP) resistance in the Amazon region.^37^,^38^ In association with severe weather changes linked to the El Niño Southern Oscillation (ENSO) climatologic phenomenon,^4^,^39^ malaria increased dramatically reaching epidemic levels between 1997 and 1999 (Figure 2). The highest annual malaria incidence in the country was reported in 1998 with more than 200,000 cases, of which about 60% and 40% were due to *P. vivax* and *P. falciparum*, respectively (Pv/Pf ratio of 1.5/1)(Figure 2). The Peruvian Amazon and the northern coast were most affected,^40^ accounting for about 45% and 40%, respectively, of the total malaria cases (approximately 530,000 cases) during the 1997–1999 epidemic period.

After the epidemic, malaria incidence in Peru dropped to 57,712 and 68,003 cases, respectively, in 2000 and 2001, followed by a slight increase and stabilization around 80,000–85,000 cases between 2002 and 2005.^41^ The decreasing contribution of *P. falciparum* cases to the total malaria cases during the period 2000–2005—as evidenced by an increase of the Pv/Pf ratio from 2.5/1 to about 5/1—may be an early effect of the implementation of a new malaria treatment policy that introduced two different artemesinin-based combination therapies (ACTs) as first-line treatments for *P. falciparum*, that is, mefloquine–artesunate (AS) in the Amazon region, and AS–SP in the northern coast.^37^ With *P. vivax* malaria, the radical treatment scheme including CQ (3 days) and PQ were also adjusted by shortening the PQ regimen from 14 to 7 days at increased daily dose (0.5 instead of 0.25 mg/kg/day).^37^

The new malaria treatment policy was implemented more quickly in the northern coast than in the Amazon, primarily because of better accessibility to malaria-endemic areas (i.e., peri-urban communities) which facilitated training and supervision of the local health facilities and staff.^42^ Test-and-treat strategies—PCD of symptomatic subjects with treatment only of confirmed malaria infections with ACT—together with implementation of community-based environmental management strategies to control the predominant vector *Anopheles albimanus* (such as the shift of rice cultivation patterns) probably contributed to the steady decline in malaria incidence observed in the northern coast from the early 2000s.^8^,^42^

**Figure 3.**
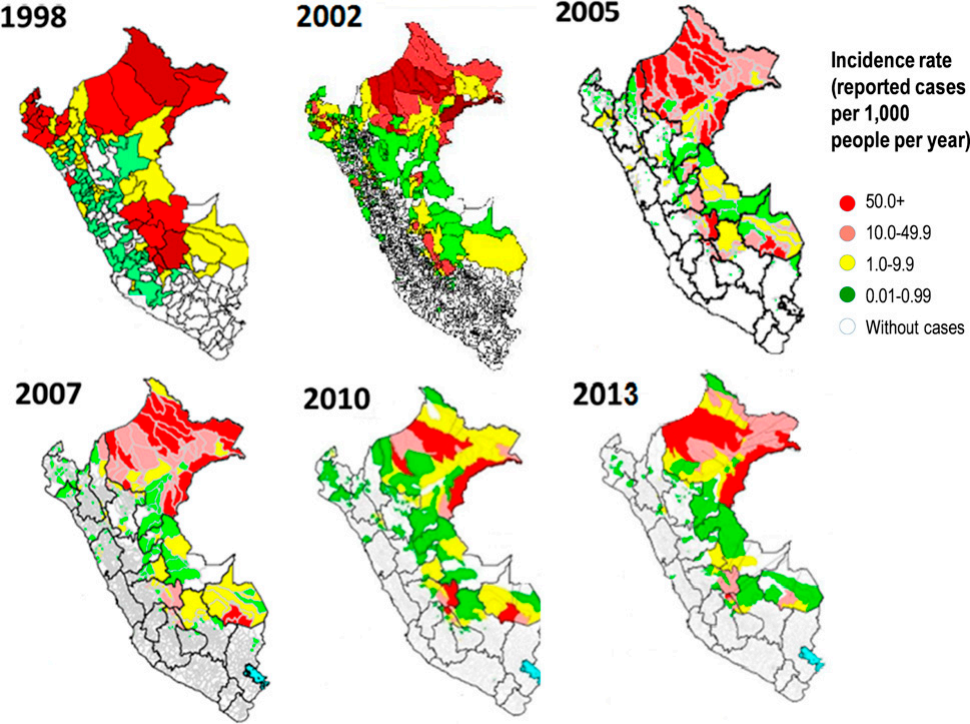
Malaria risk map of Peru based on annual incidence rates 1998–2013 (annual parasitological index [API]). Source: Peruvian Ministry of Health.

In the Amazon region, the new treatment policy took longer to implement. After 2005, the implementation process was driven by the Global Fund's Malaria Project “PAMAFRO”^43^ by increasing accessibility and quality of microscopy-based diagnosis, ensuring availability of effective antimalarials, and periodic training and supervision of local health workers, even in the most remote endemic areas. This work did not involve intervening to reduce mosquito breeding sites in communities as an alternative to the use of insecticide spraying because of lack of knowledge of where such sites lay. PAMAFRO was simultaneously applied at the border areas of Venezuela, Colombia, Ecuador, and Peru between October 2005 and September 2010, with common control strategies, including early diagnosis and treatment, active case detection (ACD) interventions in communities of high malaria risk (as determined by a set number of cases, and only carried out either under this PAMAFRO program or research conditions), mass distribution of long-lasting insecticide-treated bed nets, community participation (local health worker involvement and empowerment to implement campaign), and intensified health education and promotion campaigns.^44^–^46^ During this period, malaria declined in Peru from 87,805 reported clinical cases in 2005 to 29,339 and 23,060 cases in 2010 and 2011, respectively.^41^,^42^ Comparatively, the percentage of reduction in reported cases was higher for *P. falciparum* (85%) than for *P. vivax* (63%), thus keeping the increasing trend of the Pv/Pf ratio until reaching its maximum (11.8/1) in 2010 (Figure 3

). After almost 40 years and for the second time in the known history of malaria in Peru, the malaria incidence rate was less than 1 case/1,000 inhabitants in those 2 consecutive years (2010 and 2011) (Figure 1). The significant reduction in malaria incidence from 1998 to 2013 led to important changes in the malaria risk map in Peru; with each subsequent year, low-transmission areas predominated more and more over high- and moderate-transmission areas (Figure 3). The quantitative contributions of PAMAFRO, sociopolitical, public health practices and policies, and climate-related changes to these malaria risk map changes are not known, but remain to be explored in future work.

However, there have been alarming developments since 2011. Since then, the number of malaria cases again began to rise (Figures 2 and 3), with the total number of reported *P. vivax* and *P. falciparum* cases increasing from 20,421 to 49,745 and from 2,639 to 12,475 cases, respectively, from 2011 to 2015.^3^ One leading hypothesis to explain the rapid resurgence of malaria after 2011 is that the Peruvian NMCP was politically and financially limited so that it could not keep up with the intense control interventions in the Amazon after the PAMAFRO project ended.^6^,^42^ Because of such a rapid resurgence, other factors must have contributed including weather changes (i.e., increased rain, increased river height related to Andean snow melt related to climate change, as well as social and political disorder in Iquitos in recent years. Funds were reallocated to more pressing public health issues in the region (particularly dengue), because it was assumed that malaria was “under control,” combined with unusually heavy rains after 2011, which damage and flooding to many rural communities located along the Amazon river and all its tributaries, further worsened the situation.^42^ Political and financial commitment from the Peruvian government to combat this malaria resurgence has increased since 2015, allowing for a wider deployment of ACD interventions in the highest-risk communities. However, the impact of these efforts might be limited if strategies are not adapted to the specific local malaria epidemiology, and, importantly, commitment is not sustained. More attention to longer-term investment in elimination strategies with development and deployment of new malaria intervention tools will also be required.

## Peruvian *P. vivax*-endemic Regions

About half the total population of Peru is potentially exposed to malaria or lives in areas with ecological factors favorable to transmission,^42^ both of which are found in the northern and southern Amazon region, and in the Pacific northern coast (Table 1 ).

The northern Amazon region comprises predominantly the Loreto Department, located in the northeast of Peru and covering almost 30% of total territory in Peru and most of the Amazon region. Loreto is home to about 1 million inhabitants.^1^ Its climate is warm and humid with a heavy rainy season from November to May and a mild rainy season from June to October.^33^ Malaria transmission is perennial, peaking between February and July,^47^ but with some variations across districts. The highly anthropophilic mosquito, *An. darlingi*, is the primary malaria vector, and a recent report showed a trend in this vector for increasing outdoor biting compared with previous reports of near-equal indoor and outdoor biting.^28^ *Plasmodium vivax* and *P. falciparum* infections occur at a ratio of 4/1 mainly in people living in open or semiclosed wooden houses located in rural and remote villages along the main Amazon river and its tributaries.^7^ All age groups are at risk of infection, though adults more so than children.

Despite the fact that Loreto has been considered to be hypoendemic for malaria transmission,^16^ routine surveillance data and epidemiological studies suggest that malaria transmission in the region is highly heterogeneous, with some areas in relatively remote Amazonia having entomological inoculation rates (EIRs) rivaling those found in holoendemic regions of Africa.^19^ More than 80% of the total malaria clinical cases detected by PCD in Loreto are concentrated among only 10–20% of the total communities of the department.^6^ Moreover, cross-sectional studies using LM for malaria diagnosis have also showed that malaria parasite prevalence varied considerably across communities around Iquitos (capital of Loreto),^16^,^47^ and those differences remained or even increased when using more sensitive diagnostic tests.^16^,^48^ Indeed, the use of polymerase chain reaction (PCR) in epidemiological studies has shown that an important proportion of the total infections are asymptomatic and with low and submicroscopic (subpatent) parasite densities.^5^,^6^,^16^,^48^ Similarly, entomological studies have also shown evidence for transmission heterogeneity by finding a wide variation of EIR estimates at the microgeographical level in riverine campsites about 50 km of Iquitos and along the only paved highway in Loreto (the Iquitos–Nauta road).^19^,^26^,^49^ Among the potential explanations for the spatial heterogeneity of malaria are ecological factors that facilitate mosquito breeding and resting sites are associated with deforestation, natural and man-made water bodies (e.g., fish ponds), and surrounding secondary vegetation are among the potential explanations for the spatial heterogeneity of malaria. In addition to vector-related ecological factors, human socioeconomic differences such as poorer housing conditions (open or semiclosed houses)^50^ and outdoor economic activities (such as farming, logging, or fishing) and travel along riverine routes among endemic sites of transmission likely play an important role in the maintenance of malaria transmission, often with unexpected, stochastic patterns.^7^

The southeastern jungle of the Peruvian Amazon region, that is, the Madre de Dios Department, has long experienced intermittently epidemic and sporadic malaria, virtually all of which is due to *P. vivax*.^51^ The primary driver of malaria in this region is illegal gold mining, with attendant ecological, political, and socioeconomic change. Conventional wisdom is that malaria is repeatedly reintroduced into the region by migrants who acquired infection in endemic areas, so that small outbreaks occur, then disappear for unknown reasons. The analysis of PCD data between 2001 and 2012 (J. F. Sanchez, J. M. Vinetz, A. G. Lescano, unpublished data), showed that of 203,773 febrile cases, 30,811 (15%) were confirmed cases of malaria, all due to *P. vivax*. The 10 *P. falciparum* cases identified during this period were likely imported from Brazil. Health facilities located in areas of intense illegal gold mining reported 30-fold more malaria cases than those in nonmining areas (ratio = 31.54, 95% confidence interval [CI] = 19.28, 51.60); health facilities located more than 1 km from the InterOceanic Highway reported significantly more cases than those within this distance (ratio = 16.20, 95% CI = 8.25, 31.80).

Transmission of malaria in Madre de Dios is unstable and geographically heterogeneous. The primary malaria vector is *An. darlingi*, but to date it has proven difficult to identify breeding sites of this mosquito species within environmental contamination and degradation related to human settlements and mining-related chemical discharges in the malaria-affected areas. *Anopheles* (*Nyssorhynchus*) *benarrochi* B and *Anopheles* (*Nyssorhynchus*) *rangeli* were also found among anopheline specimens obtained and analyzed in 2011 by human landing catch, Shannon and CDC traps (Centers for Disease Control and Prevention, Atlanta, GA) from the malaria endemic localities of Santa Rosa and San Pedro in Madre de Dios Department, Peru; neither of these potential secondary vectors were found to be positive for *Plasmodium* infection by cytochromb b (CytB)-PCR, although the sample size was relatively small.^52^ Research findings in Madre de Dios highlight the unpredictable nature of malaria transmission in areas of Peru where public health policies are difficult to implement, and only control measures based on PCD have been carried out. These observations underscore the importance of continued stochastic reintroductions of malaria, that timely, surveillance-based interventions are needed to control malaria in neglected and politically challenging regions, and the need for new tools for monitoring and predicting newly emerging malaria in areas where anophelism remains, for example, in illegal gold mining camps.

The northwestern coast, which includes Tumbes, Piura, Lambayeque, and La Libertad departments, has historically been the second most important region for malaria transmission after the Amazon rainforest.^4^ This region is characterized by the presence of coastal valleys conducive to propagation of anopheline vectors (primarily *An. albimanus*) as well as by human migration for labor-intense agricultural activities.^53^ The region typically has hot and humid summers with moderate rains from December to March and winter seasons from April to November with cool and dry days. However, this seasonal climate pattern has changed in the past by the ENSO phenomenon,^39^ with torrential rains and strong winds causing flooding and landslides, events that have been associated with malaria outbreaks (e.g., malaria epidemic in 1997–1998).

Malaria has significantly declined in north-coastal Peru over the past decade.^3^ Currently, reported malaria cases are exclusively *P. vivax* and concentrated in a few localities of Piura and Tumbes.^3^ Transmission occurs in epidemic and sporadic patterns between February and June, the months of highest density of the main vector, *An. albimanus*.^53^ The most affected people live in peri-urban localities in close proximity to irrigated fields and irrigation canals which enable anopheline mosquitoes to propagate and survive.^20^,^53^ Although houses in those localities have complete walls (closed houses), they are mainly made of poor materials that do not exclude mosquitoes.

## *Plasmodium vivax* Malaria in Peru

Recent years have seen the increased recognition that *P. vivax* malaria may present as a severe disease according to newly published World Health Organization (WHO) criteria.^54^–^57^ Severe malaria has been observed in Peru, both in the northern coast^58^ and related to travelers who presented in Lima (a nonendemic area) with severe malaria due to *P. vivax* (and *P. falciparum*) acquired in known malarious regions.^9^ Severe vivax malaria in Peru appears to have similar characteristics compared with elsewhere in Amazonia,^59^ but appears to be less common in Amazonia than in India.^60^ In the Loreto region, more than two-thirds of cases of severe malaria arriving at referral hospitals are due to *P. vivax* and belong to Group I of the new WHO classification, meaning that, because of the possibility of *P. falciparum* coinfection (without parasitological proof),^61^ such cases must be treated with intravenous AS in addition to standard *P. vivax* therapy. Although such cases present with profound prostration, the level of consciousness is generally preserved. Consistent with this observation, recent autopsy information from Brazil indicates that *P. vivax* is not found in brain in patients with coma.^62^ Limitations of studies of severe vivax malaria include lack of investigation of comorbid illness or coinfection, and their retrospective nature. Further, mechanisms of severe *P. vivax* malaria pathogenesis, whether from the host response or parasite virulence mechanisms, remain to be explored, particularly with regard to pulmonary and multiorgan failure syndromes.

## Detection of *P. vivax* Infection

In Peru, governmental policy dictates that LM remains the main approach to diagnose acute malaria and guide appropriate treatment.^63^ The Peruvian Ministry of Health has established a strong National Network of Public Health laboratories classified into four levels: local, intermediate, regional, and national level. This network is designated to perform both diagnosis and quality control of thick blood smears,^64^ according to international norms published by the WHO.

Microscopy is routinely performed on samples from all febrile cases who present to health facilities and/or hospitals, local and intermediate laboratory levels, respectively, with signs and symptoms suggestive of acute malaria. In 2015 in Loreto, 321,723 thick blood smears were read at health facilities as part of this PCD, and 48,987 thick blood smears were read as part of reactive ACD, from collaterals, individuals who live in close proximity to passively detected cases (Directorate of Public Health Reference Laboratory, Iquitos, Peru, personal communication). After PAMAFRO ended in 2010, ACD activities carried out by the Regional Health Office (DIRESA) substantially declined in the region.

Directly related to current resurgent *P. vivax* and *P. falciparum* malaria in the Loreto Department of Peru, access to timely and effective malaria diagnostics remains a challenge in the region. During the PAMAFRO project, the coverage of the National Network of Public Health laboratories increased with purchase and deployment of new microscopes combined with intensive training of health personnel.^46^ There are still areas where, because of logistical challenges, other diagnostic tools should be used, such as point-of-care deployable rapid diagnostic tests (RDTs). RDTs were introduced in Peru in 1998 as an alternative to microscopy in remote areas of the Peruvian Amazon. However, the clinical performance of RDTs has not been consistently useful in Peru. RDTs were last reported to be used in Loreto for confirmatory diagnosis in 2006, within the PAMAFRO project, with an unacceptable performance (sensitivity of 77% and 54% for non-falciparum and *P. falciparum*, respectively); results were observed to be even worse when parasitemia was less than 100/μL on LM^65^ In addition, many false-negative RDTs related to histidine-rich protein (HRP)-2 have been observed. Published data from Peru indicate that *P. falciparum* field isolates commonly lack the *pfhrp2* gene (the main target used by previously used RDTs) limiting further the use of some RDTs.^66^ Another study with RDTs in this region, also showed cross-reactions of *P. vivax* samples with the Pf-pLDH or PfHRP2 tests; *P. falciparum*-positive samples with high parasitemias cross-reacted with Pv-pLDH.^23^ Based on these observations, the ideal RDT to be used in remote areas of the Peruvian Amazon should target Pf-pLDH and Pv-pLDH separately in the same device, with good heat stability and better sensitivity. Currently, these RDTs are not able effectively to identify very low parasitemia—either of *P. vivax* or *P. falciparum*—to identify asymptomatic reservoirs of transmission.

Serological tests, unlike LM and RDTs, cannot be used for the diagnosis of acute malaria because antibodies can persist for months or years after infection has resolved.^67^ However, combining serological responses with geospatial analysis enables the accurate identification of hotspots for targeting with appropriate interventions.^6^,^11^ In two studies with contrasting epidemiological settings of Peru, the use of PCR to detect current malaria infections due to *P. vivax* and *P. falciparum*, and serology to identify past malaria exposure to both species, have repeatedly proved their added value in accurately understanding the local epidemiology, determining risk factors for malaria, and describing spatial heterogeneity in transmission.^6^,^11^ In a third study conducted in the northern coast, spatial analysis using specific IgG responses to PvMSP-119 and PvAMA-1 of individuals enrolled in a cross-sectional survey proved to be reliable for detecting hotspots of clinical malaria cases detected by PCD in the following year.^42^ A number of knowledge and technical gaps need still to be addressed for developing and/or optimizing serological tests for programmatic surveillance.^68^ These research gaps are larger for *P. vivax* than for *P. falciparum*, since less information is available about *P. vivax* antigens for use in serosurveillance.^68^

## Treatment Efficacy for *P. vivax* Disease and for Preventing Recurrent Episodes

The widespread use of CQ plus PQ, in combination, may be the reason for the few, isolated cases of CQ-resistant *P. vivax* (CQRPV) reported in Peru. Indeed, Peru is one of the few countries in the Americas with reports of CQ resistance,^69^ but with no new data reported since 2003. A 28-day drug efficacy trial in the Amazon region and the northern Coast between 1998 and 2001, including 242 *P. vivax*-infected individuals who received CQ alone (25 mg/kg, over a 3-day period), found four individuals with recurrence of *P. vivax* parasitemia on days 21 and 28 after treatment. All were from the Amazon, and in two of them, CQRPV was confirmed by measuring drug levels at the time of recurrence.^38^ More recently, a drug effectiveness study also in the Amazon found that four of 540 patients treated with the CQ–PQ combination had a symptomatic recurrence of *P. vivax* parasitemia within 35 days of treatment; however, in only one of them, the recurrence occurred against normally therapeutic blood levels of CQ, suggesting a probable case of CQRPV.^70^

Evaluating the contribution of relapses in the *P. vivax* burden of endemic regions, as well as assessing the efficacy of drugs to prevent *P. vivax* relapse, is challenging because a recurrent *P. vivax* infection cannot easily be distinguished between recrudescence (i.e., failure to treat the initial infection), reinfection (i.e., new infection), or relapse (i.e., hypnozoite reactivation).^10^ Nevertheless, three studies conducted in the Peruvian Amazon with strict follow-up of individuals with confirmed *P. vivax* infections after receiving radical cure treatment, showed that *P. vivax* recurrences are relatively common in the Amazon region. The first study conducted between 2006 and 2008 was a 6-month follow-up of patients who were randomly assigned to treatment schemes including CQ plus three different PQ regimens (5 days of PQ at 0.5 mg/kg/day, 7 days of PQ at 0.5 mg/kg/day, and 14 days of PQ at 0.25 mg/kg/day).^24^ Schemes with 7 and 14 days of PQ did not show significant differences in recurrence rates (10% versus14%) during the follow-up period, but they were more effective in preventing them than the scheme with only 5 days (28.4% of recurrences). The second study was a 2-year cohort of *P. vivax*-infected individuals from 29 Amazonian communities (2008–2011) aimed to evaluate the efficacy of current national treatment of the prevention of recurrent infections. Of 270 individuals who completed the 2-year follow-up, 53% (144) had *P. vivax* recurrent infections, most of them (70%) had several with a median of three recurrences (range, 211 recurrences).^71^ The third study was a phase 2b multicenter clinical trial (2011–2013) that followed up 136 infected individuals in the Peruvian Amazon for 6 months to assess dose response and safety of single-dose TQ plus CQ, PQ plus CQ, and CQ alone for *P. vivax* radical cure.^13^ Findings showed high efficacy of regimens including single-dose TQ of 300 mg and 600 mg with, respectively, 81% and 84% recurrence-free efficacy at 6 months, in comparison with 59% and 12% recurrence-free efficacy at 6 months for recommended WHO scheme based on 14-day PQ plus CQ and CQ alone, respectively. Adverse events were similar between treatments, and there was a dose-dependent effect on symptomatic homologous strain relapse (but not heterologous strain reinfection); 50% of recurrent infections were homologous (i.e., same molecular genotype).^14^

## Genetic Diversity and Population Structure of *P. vivax*

Population genetics studies in the Peruvian Amazon have revealed high heterogeneity in *P. vivax* transmission, with low or high population diversity at community level, and high diversity among populations. Recent large-scale whole genome surveys of *P. vivax* from all endemic regions confirm these findings, and demonstrate that Peruvian isolates have generally diverged from global populations except for some imported cases from Brazil.^72^,^73^ Studies conducted in communities along Iquitos–Nauta road, or with high mobility of inhabitants, showed high diversity accompanied by a considerable proportion of polyclonal infections and high multiplicity of infection (MOI) (i.e., average number of parasite lineages per infected individual). Study findings in 2006 in the riverine community Mazan (north of Iquitos), where people have high mobility because of their economic activities (farming, logging, and fishing), and in peri-Iquitos-urban Moronococha, both showed high heterozygosity (*H*_*e*_ 0.66 and 0.69, respectively) and high proportion of polyclonal infections (44–70%).^74^ Similar results were found in another peri-Iquitos village, Zungarococha, between 2003 and2004, within urban areas in Iquitos and in rural communities along the Iquitos–Nauta road.^30^,^75^ A recent longitudinal study conducted over 25 different villages (among five study areas) confirmed these findings, showing that study areas along the highway from Iquitos to Nauta (cluster A2 and A3) had higher MOI (1.5–2) than isolated communities with low mobility (cluster A1, A4, and A5). However, *H*_*e*_ was high in all communities (*H*_*e*_ 0.66–0.76).^76^ On the other hand, in San Carlos, a relatively small and isolated village, at 10 km from the Iquitos–Nauta road, genetic diversity and MOI were constrained (*H*_*e*_ = 0.49, MOI = 1.1).^71^,^74^ Similar patterns were reported in other peri-Iquitos villages: Fray Martin, Santa Rita, San Jose de Lupuna, and San Pedro, rural communities at about 3–7 km north of Iquitos, on the other bank of Nanay river, only accessible from Iquitos by boat.^74^ These observations collectively suggest that in this setting of isolated settlements, where inhabitants carry out most of their activities in and around their communities and seldom visit or travel to other places, *P. vivax* diversity seems to be restricted with few polyclonal infections and low MOI. Although *P. vivax* populations in Iquitos and neighboring areas are diverse, there is strong genetic differentiation among geographically isolated sites, suggesting that transmission tends to be local and clustered.

Genetic analysis of *P. vivax* infections from four of the total 29 communities (2008–2011) included in the previously mentioned 2-year cohort study was done to evaluate the efficacy of current national treatment of the prevention of recurrent infection,^71^ and showed that 82% of recurrent infections carried at least one different allele from those at day 0 (day of treatment). However, a different pattern was found in other communities (e.g., San Carlos) where most recurrent infections were caused by similar haplotypes, suggesting true relapse rather than reinfection.^17^,^29^,^75^,^77^

The high genetic diversity of *P. vivax* in Peru might be considered unusual given the relatively low transmission intensity in the region, and probably relates to accumulation of genetically heterogeneous hypnozoites over time, a biological feature of *P. vivax* that makes it impossible to definitively differentiate relapse from reinfection.^78^ The high level of genetic diversity of *P. vivax* in Peru (and elsewhere), as demonstrated in recent global genomic surveys^72^ as well as by the use of other molecular markers,^30^ is a major feature of *P. vivax* biology, and is key for understanding the transmission epidemiology of this malaria parasite.^79^

## Vector Biology

*Anopheles darlingi* remains the most widespread, aggressive, and intractable malaria vector in Amazonia, including Peru. Invasion of both newly created (anthropic) and natural breeding sites is often swift and likely linked to high adaptability/plasticity.^80^,^81^ Where fishponds are the main breeding site, *An. darlingi* abundance^82^ and fishpond presence near human habitation are significantly associated with increased numbers of malaria cases. The identification of such new risk multipliers reveals lacunae in our understanding of *An. darlingi*'*s* success, that is, patterns of dispersal, survival, and genetic adaptability. Anopheline survival, one of the key factors in determining vectorial capacity and spatiotemporal transmission patterns, is understudied in *An. darlingi* in the field.

To our knowledge, only one study in Peru (along the Iquitos–Nauta Highway) has characterized and analyzed breeding sites in relation to malaria risk.^79^ Heterogeneity of *An. darlingi* feeding behavior is well documented and includes endophagy, exophagy, anthropophily, and opportunism, depending on habitat (landscape), host availability, and quality of breeding sites. Behavioral variability has been posited as an explanation for *An. darlingi*'s broad ecological success, but we have found (J. Conn and others, unpublished data) genetically differentiated exophagic and endophagic *An. darlingi* populations. A critical knowledge gap—currently being experimentally addressed in ongoing studies—is whether these *An. darlingi* populations differ in vector competence and can be specifically targeted for more effective vector control and elimination.

## Experimental Studies of *P. vivax* Transmission to *An. darlingi* in Peru

It has long been established that human host immune factors modulate *P. vivax* transmission from humans to mosquitoes^83^; experimental studies in the Peruvian Amazon using wild-caught, F1-generation *An. darlingi* mosquitoes have confirmed and extended these observations.^84^,^85^ Understanding mechanisms by which the human host response may impair parasite infectivity for mosquitoes has direct implications for the development of transmission-blocking vaccines. Experiments using standard membrane feeding assays found that only about half (52/102) of *P. vivax* parasitemic subjects successfully infected mosquitoes.^78^,^79^ Transmitters were more likely to have microscopically visible gametocytes, higher parasitemia, and, in terms of basic clinical parameters, a slower pulse rate than nontransmitters. Quantitative assessment of gametocytemia, using the first-reported real-time reverse transcriptase Pvs25 PCR assay to quantify *P. vivax* gametocytes, was significantly and positively correlated with oocyst counts. These experiments were the first to establish a system of determining transmission patterns in experimental infection of outbred natural neotropical malaria vectors in the Amazon region and concluded that *P. vivax*-infected subjects inefficiently infected outbred F1 *An. darlingi* mosquitoes. The results raised the possibility that some degree of naturally occurring transmission-blocking immunity is present on a population basis in the Peruvian Amazon, an area of low intensity of malaria transmission.^84^,^85^

Toward the goal of determining mechanisms that modulate the efficiency by which *Plasmodium* spp. infect *An. darlingi* mosquitoes, recent reports describe the establishment of laboratory colonies of *An. darlingi* in Peru.^86^ The availability of these colonies sets the stage for further experimental study of the transmission biology of malaria in Amazonia, both with regard to *P. vivax* and *P. falciparum*, whether from infected humans to mosquitoes, or conversely with regard to obtaining *P. vivax* sporozoites for use in in vitro experimental infections, and potentially in vivo in controlled human challenge infections, as is being carried out in Australia and Colombia.^87^ Peruvian Ministry of Health in Loreto approval has been obtained to carry out direct feeds on malaria-infected individuals as well, the safety of which is enhanced by using the colonized mosquitoes (currently stable and propagating well at generation 40) which do not have known transovarially transmitted pathogens. Therefore, in Peru, there is the infrastructure and capacity to carry out future experiments to more precisely determine the infectivity efficiency of different *P. vivax* parasitemic individuals for *An. darlingi*, for example, comparing symptomatic (who have higher and patent parasitemia) and asymptomatic (who have lower parasitemias, whether patent or subpatent) subjects.

## Conclusions: Key Public Health Challenges for Controlling and Eliminating *P. vivax* Infection

Strategies both to control and eliminate *P. vivax* in Amazonia will have to take into account the complex interactions of parasite biology (*P. vivax* liver-infecting dormant hypnozoites), human behavior, and highly variable environments that drive changes in mosquito vector behaviors. These factors have led to continued low transmission and residual malaria in Peru, and underlie challenges to the sustainability of conventional malaria control strategies, as illustrated by repeated malaria resurgences over decades whenever control measures are not sustained. The complexity of Amazonian malaria is augmented by intense human movement related to work and social interactions, which combined with asymptomatic infections lead to “silent” reservoirs of malaria parasites moving across space and time and maintain endemic malaria transmission.

To overcome these challenges in coming years, research should focus on development, evaluation, and deployment of new molecular and biomarker tools for the rapid and efficient identification of individual with hypnozoites, and asymptomatic, subpatent human reservoirs of continuing transmission. Epidemiology studies need to focus on and target human behaviors that serve to maintain and move malaria parasites around endemic regions. Further, as all effective malaria control measures in the past have intervened against the vectors, understanding the plasticity, breeding, host tropisms, and genetic factors that drive *An. darlingi*-transmitted malaria in Amazonia.

Finally, it is important to emphasize that academic research, such as described herein, has important potential to inform public health officials at local, national, and international levels. In Peru, such interactions have increasingly taken place at these different levels, and academic researchers from Peru have been participating in meetings with the Loreto Ministry of Health, the NMCP, and the U.S. Agency for International Development–organized Amazonian Malaria Initiative in which the Pan American Health Organization is a partner. In venues such as these, it is important for data-driven research to inform policy-making toward the goal of regional elimination, a major goal in Peru. Developing and maintaining policies toward making long-term commitments to malaria elimination means incorporating new data and tools emerging from field- and laboratory-based studies toward novel elimination strategies.

## Figures and Tables

**Table 1:** Characteristics of malaria transmission in Peruvian endemic regions

Characteristics	Northern Amazon	Southern Amazon	Northern coast
Period of transmission	Perennial transmission with marked seasonality	Seasonal, stochastic	Seasonal transmission
Environmental conditions	Tropical rainforest, heavy rains (November–April)	Tropical rainforest, heavy rains (December–April)	Dry desert area, rainfall (December–March) influenced by ENSO
Other ecological conditions	Deforestation	Deforestation	Rice farming
Vector	*Anopheles darlingi*, *Anopheles benarrochi*	*An. darlingi*, *An. benarrochi*	*Anopheles albimanus*
Parasite	*Plasmodium vivax* (80%), *Plasmodium falciparum* (20%)	*P. vivax*	*P. vivax* (99%), *P. falciparum* (< 1%)
Risk areas	Rural, peri-urban	Rural	Urban, peri-urban
House characteristics	Open houses without walls or rudimentary walls	Open houses without walls or rudimentary walls	Closed houses with completed walls
Economical activities	Subsistence-scale agriculture, logging, fishing, hunting	Illegal gold mining	Commerce, fishing, agriculture
Accessibility to health services	Limited	Limited	Accessible

ENSO = El Niño Southern Oscillation.
